# Supplementary material for: Horizontal transfer of the rfb cluster in Leptospira is a genetic determinant of serovar identity
Source: Life Sci Alliance. 2022 Dec 9;6(2):e202201480. doi: 10.26508/lsa.202201480 (PMC9736851; doi:10.26508/lsa.202201480)
Supplement: Supplementary file 1 [file LSA-2022-01480_Supplemental_Data_1.docx]

**“Horizontal transfer of the *rfb* cluster in *Leptospira* is a genetic determinant of serovar identity” by Nieves C et al.**

**Supplemental Material**

- Supplemental Figure S1 – ANI

- Supplemental Figure S2 – POCP

- Supplemental Figure S3 – Pangenome analysis

- Supplemental Figure S4 - Distribution of transposase genes in chromosome 1 from *L. noguchii* strains.

- Supplemental Figure S5 – GC-content of chromosome 1 in different *Leptospira* species; note the systematic decrease correlated to the location of the *rfb* cluster

- Supplemental Table S1 – ANI *L. noguchii* strains

- Supplemental Table S2 – Functional mapping of strain-specific genes

- Supplemental Table S3 – Plasmid repertoire of the *L. noguchii* strains sequenced in this study, and of other *Leptospira* species

- Supplemental Table S4 – Strains used to perform *L. noguchii* phylogeny analyses

- Supplemental Table S5 – *Leptospira* species and strains used to perform genetic analyses of the *rfb* cluster

- Supplemental Table S6 – *rfb* genes were used to search orthologous protein sequences: Blast best hits’ accession numbers are listed.

- Supplemental References.

All supplemental figures are shown below, and also uploaded as separate individual files. Supplemental tables and files (gbk from annotation with Prokka) are attached separately.


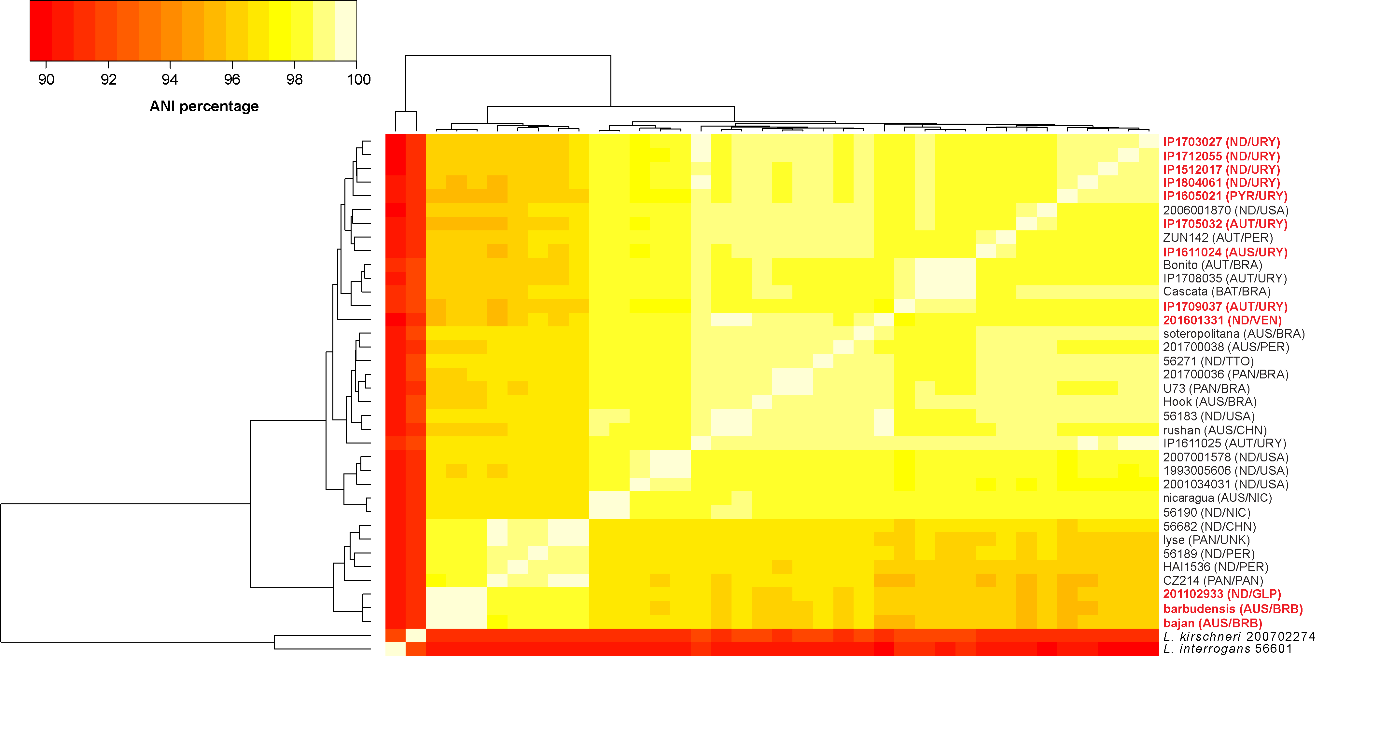


**Supplemental_Fig_S1. Average Nucleotide Identity (ANI) among *L. noguchii* strains with reported whole genome sequences.** *L. interrogans* and *L. kirschneri* were included as reference of distinct species. The ANI percentages are depicted as colors of the square matrix elements, according to the scale shown in the upper left insert. The names of the twelve strains sequenced in this study are highlighted with red fonts. Clustering shown on the left side (and upper side, symmetric) of the matrix table, was performed by GET_HOMOLOGUES version 20190411 (Contreras-Moreira and Vinuesa 2013). Each strain’s serogroup (AUS=Australis; AUT=Autumnalis; BAT=Bataviae; ND=not determined; PAN=Panama; PYR=Pyrogenes), and the country from where they were obtained (BRB=Barbados; BRA=Brazil; CHN=China; GLP=Guadeloupe; NIC=Nicaragua; PAN=Panama; PER=Peru; TTO=Trinidad & Tobago; USA=United States of America; URY=Uruguay; UNK=unknown; VEN=Venezuela) are indicated in parentheses.


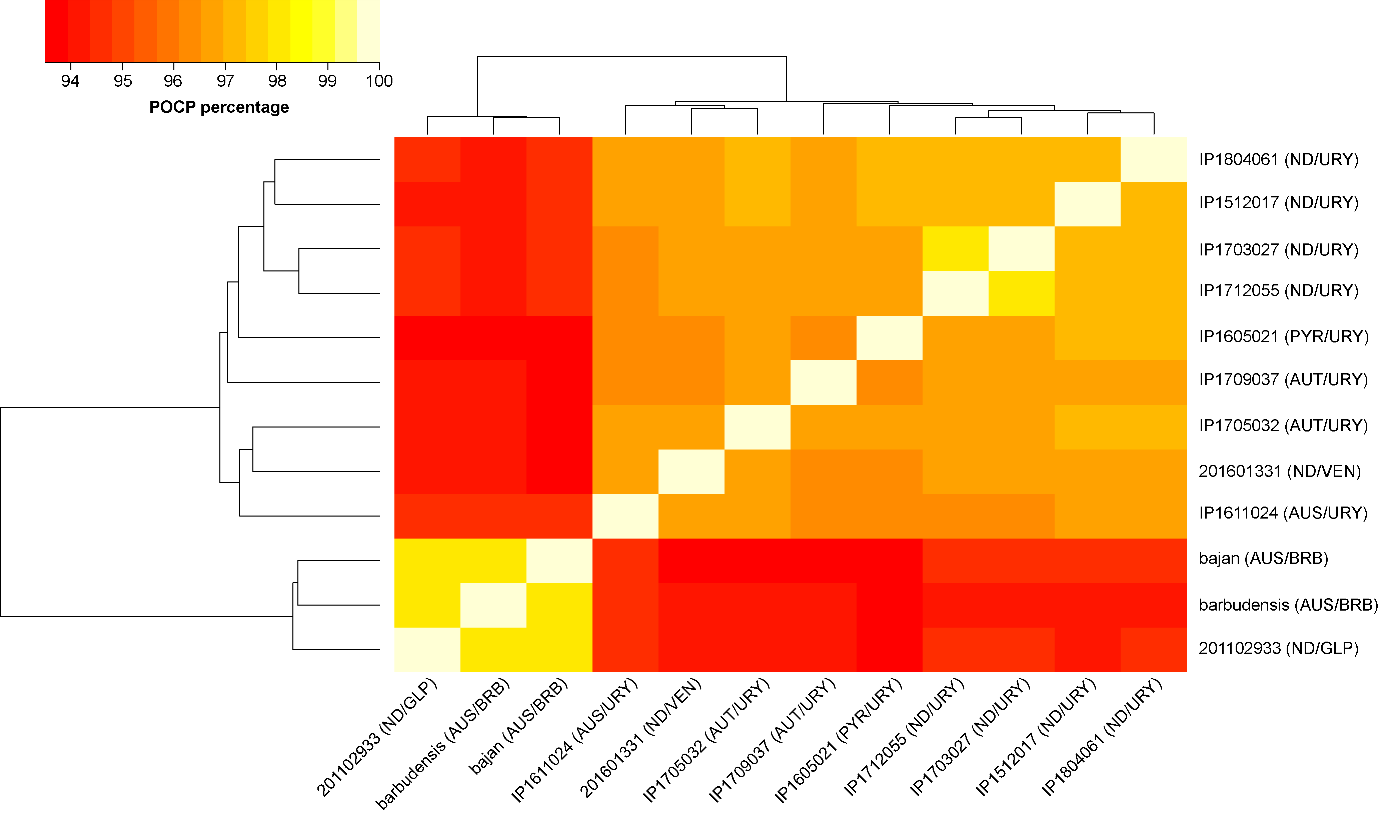


**Supplemental_Fig_S2. Percentage of Conserved Proteins (POCP) among *L. noguchii* strains.** POCP percentages are represented as colors of the square matrix elements, according to the scale shown in the upper left insert. The names of the twelve sequenced strains are indicated on the right and bottom. Clustering shown on the left and upper sides of the matrix, was performed by GET_HOMOLOGUES version 20190411 (Contreras-Moreira and Vinuesa 2013). Strain names identifiers as in Supplemental Fig. S1.


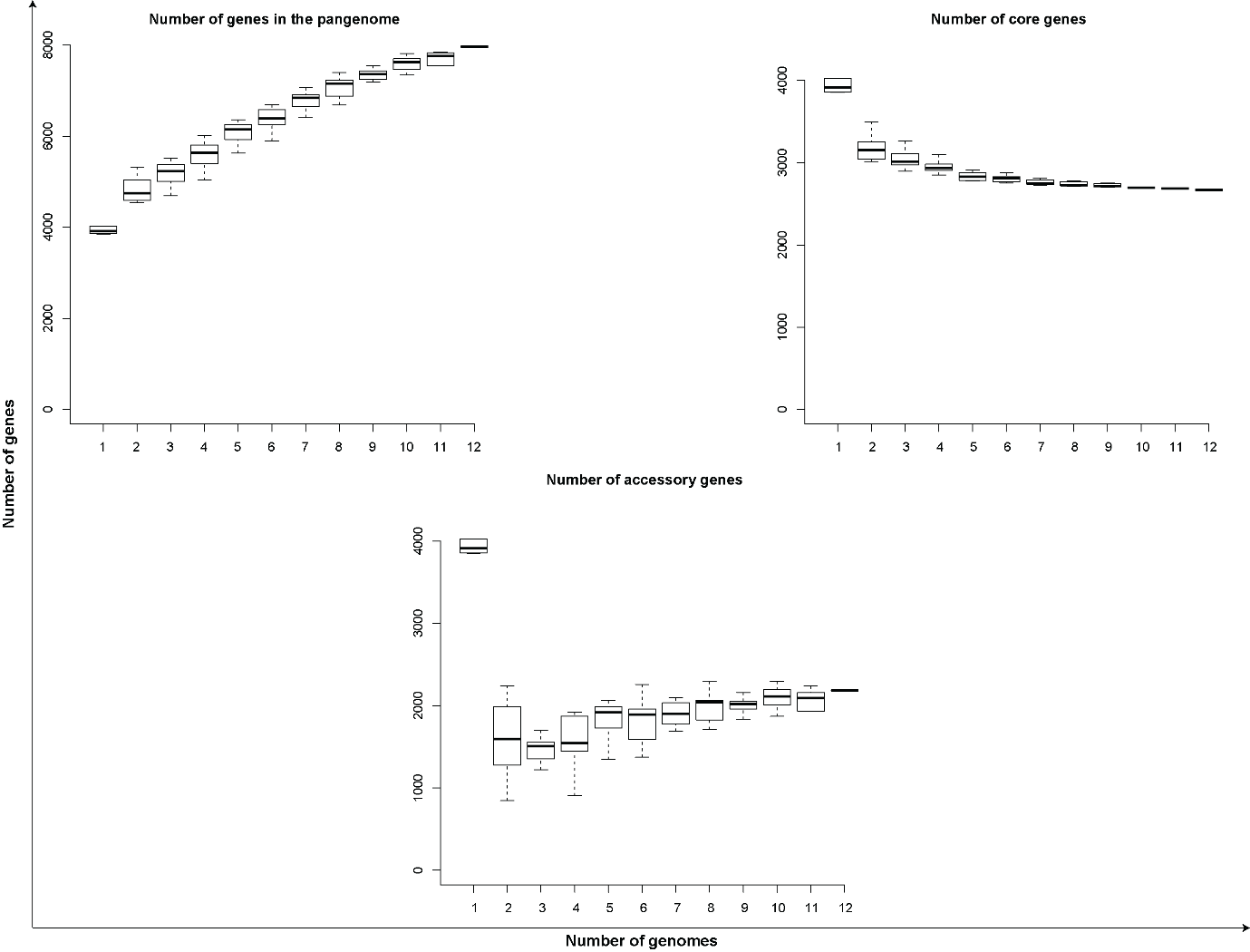


**Supplemental_Fig_S3. Pangenome profiling of the twelve *L. noguchii* strains sequenced in this study.** Calculations and plots were performed using Roary 3.11.2 (Page et al. 2015). The curve observed in the pangenome plot was used to fit a calculated curve according to Heaps’ law (Tettelin et al. 2008), n = κN^γ^, considering a total of 7963 genes (n) in the pangenome *vs* 12 genomes (N) included, the observed curve allows to fit non-linearly κ = 1610.8 and 1-γ = α = 0.36. α <1 indicates an open profile.


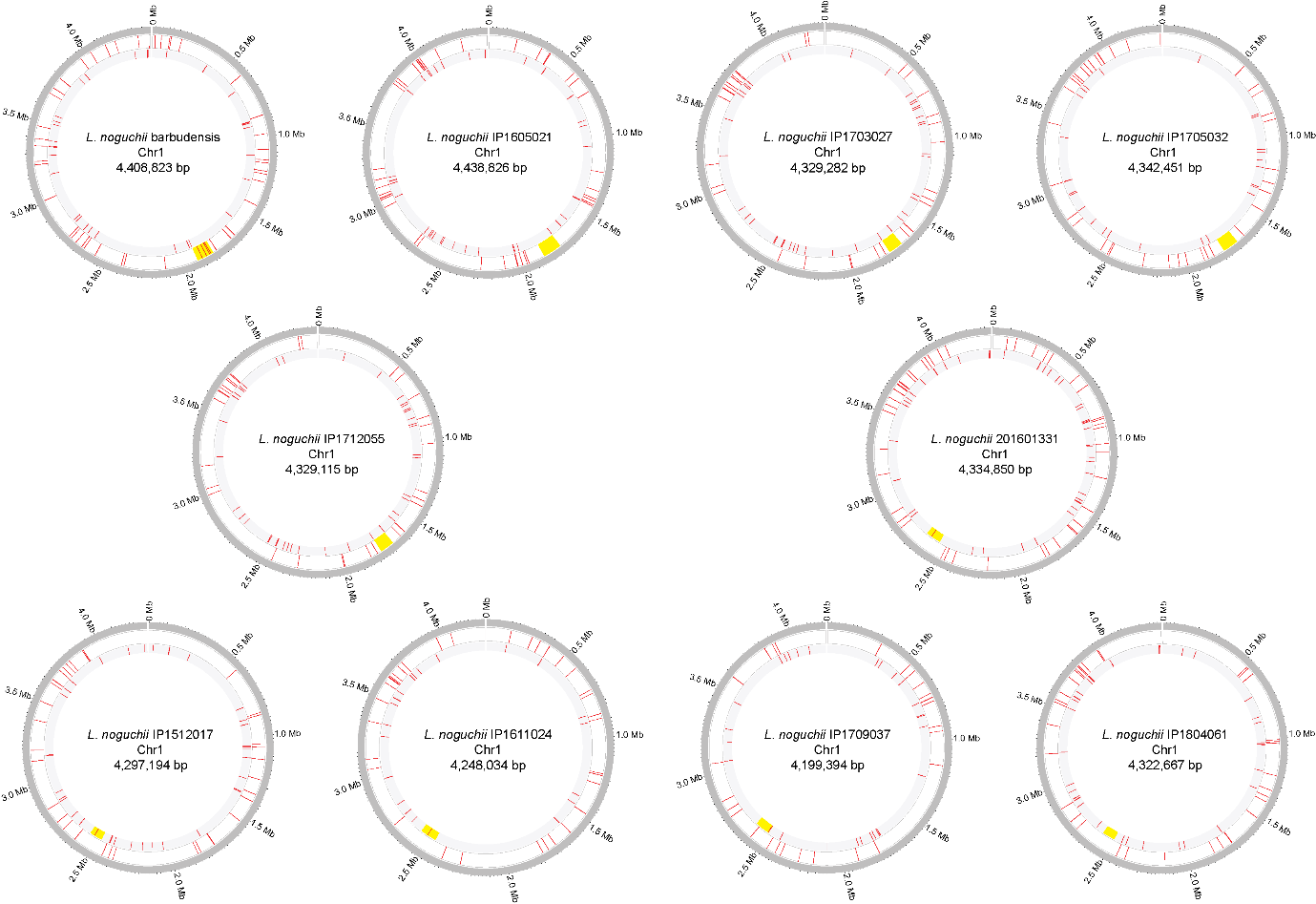


**Supplemental_Fig_S4. IS/transposase genes distribution across chromosome 1 from *L. noguchii* strains sequenced in this study**. Transposase and transposase-like genes are depicted as red lines; the complementary and leading strands are represented respectively on the inner and the outer circle positions. The *rfb* cluster is shown as yellow blocks. This graphical representation was produced with the online version of shinyCircos (<https://venyao.xyz/shinycircos/>) (Yu et al. 2018).


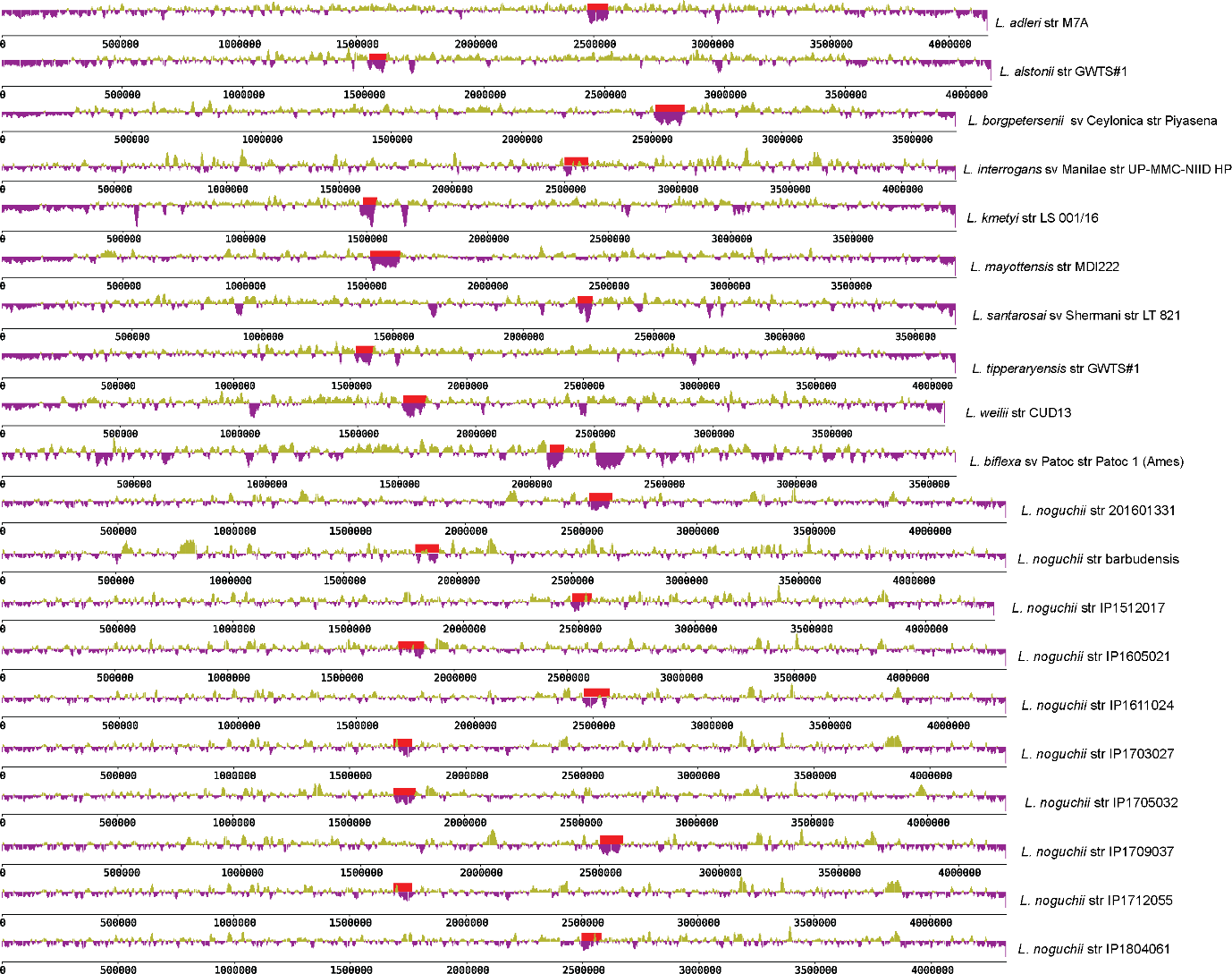


**Supplemental_Fig_S5. GC-content across chromosome 1 from different *Leptospira* species.** Chromosomes 1 from different *Leptospira* species are laid down, with the *rfb* cluster depicted as a red bar on top of each one. The GC-content (calculated every 1000 bp) was plotted in colored curves using DNAplotter (Carver et al. 2009), with purple highlighting a decrease in its percentage, and green an increase compared to the average found in the whole chromosome 1.

**Supplemental References**

Carver T, Thomson N, Bleasby A, Berriman M, Parkhill J. 2009. DNAPlotter: circular and linear interactive genome visualization. *Bioinformatics* **25**: 119-120.

Contreras-Moreira B, Vinuesa P. 2013. GET_HOMOLOGUES, a versatile software package for scalable and robust microbial pangenome analysis. *Appl Environ Microbiol* **79**: 7696-7701.

Page AJ, Cummins CA, Hunt M, Wong VK, Reuter S, Holden MT, Fookes M, Falush D, Keane JA, Parkhill J. 2015. Roary: rapid large-scale prokaryote pan genome analysis. *Bioinformatics* **31**: 3691-3693.

Tettelin H, Riley D, Cattuto C, Medini D. 2008. Comparative genomics: the bacterial pan-genome. *Curr Opin Microbiol* **11**: 472-477.

Yu Y, Ouyang Y, Yao W. 2018. shinyCircos: an R/Shiny application for interactive creation of Circos plot. *Bioinformatics* **34**: 1229-1231.
