# Supplementary material for: Horizontal transfer of the rfb cluster in Leptospira is a genetic determinant of serovar identity
Source: Life Sci Alliance. 2022 Dec 9;6(2):e202201480. doi: 10.26508/lsa.202201480 (PMC9736851; doi:10.26508/lsa.202201480)
Supplement: Supplementary file 9 [file LSA-2022-01480_Supplemental_Data_3.zip › Supp_Data_2-rfb_sequences/README_caption.rtf]

Supplemental_Data_2: DNA sequences of rfb clusters (listed in Table S6), in GenBank plain text format with header CDS annotations as obtained from Prokka
